# Evaluating the validity and performance of a modifiable prediction model to predict new long-term opioid use in prescription drug monitoring programs

**DOI:** 10.1093/jamiaopen/ooag192

**Published:** 2026-09-30

**Authors:** Iraklis Erik Tseregounis, Eugene Shin, Stephen G Henry, Chris Delcher

**Affiliations:** Division of General Internal Medicine & Bioethics, Department of Internal Medicine, University of California, Davis, Sacramento, CA 95817, United States; Center for Healthcare Policy & Research, University of California, Davis, Sacramento, CA 95817, United States; Institute for Pharmaceutical Outcomes & Policy, Department of Pharmacy Practice and Science, College of Pharmacy, University of Kentucky, Lexington, KY 40536, United States; Division of General Internal Medicine & Bioethics, Department of Internal Medicine, University of California, Davis, Sacramento, CA 95817, United States; Center for Healthcare Policy & Research, University of California, Davis, Sacramento, CA 95817, United States; Institute for Pharmaceutical Outcomes & Policy, Department of Pharmacy Practice and Science, College of Pharmacy, University of Kentucky, Lexington, KY 40536, United States

**Keywords:** clinical decision support, prescription drug monitoring programs, opioid prescribing, long-term use, external validation

## Abstract

**Objective:**

Prescription drug monitoring programs (PDMPs) offer a promising data source for the development of clinical decision support (CDS) systems to guide opioid prescribing, but most PDMP agencies lack capacity for in-house model development—instead relying on third-party tools. This study aims to externally validate a PDMP-based model, developed using 2018 California PDMP records, to predict new long-term use among Kentucky opioid-naïve patients and compare its performance to Kentucky-specific machine learning (ML) models.

**Materials and Methods:**

We identified opioid-naïve Kentucky residents who filled an index pill opioid prescription between 2021 and 2023 (development cohort: 2021-2022; validation cohort: 2023). The California model was updated via logistic calibration and evaluated against 5 newly developed Kentucky ML models (least absolute shrinkage and selection operator [LASSO], elastic net, deep neural network, random forest, XGBoost) using discrimination and calibration metrics.

**Results:**

Among 985 317 opioid-naïve patients, 33 298 (3.4%) transitioned to new long-term opioid use. The updated California model achieved excellent discrimination (area under the receiver operating characteristic curve [AUROC]: 0.919 [0.915-0.923]) and was calibrated up to the highest risk levels (calibration intercept: 0.010, [−0.021, 0.041]; slope: 0.997, [0.979, 1.014]), with negative predictive values exceeding 0.99. Performance was comparable to Kentucky-specific ML models, including the highest performer XGBoost (AUROC: 0.930 [0.926-0.933]). All models struggled with low positive predictive values (0.15-0.19).

**Conclusion:**

A California PDMP-derived prediction model transported successfully to Kentucky with minimal updating. Its strong comparative performance provides a feasible foundation for PDMP agencies to develop transparent, in-house CDS systems with potential clinical implications for safer and more individualized opioid prescribing.

## Introduction

Opioid use disorder and overdose remain significant public health issues in the United States. The Centers for Disease Control and Prevention (CDC) opioid prescribing guidelines, released in 2016^1^ and revised in 2022,^2^ emphasize limiting the number of patients who receive a new opioid prescription and continue opioids for the long term to reduce the risk for opioid-related harms. Initially, these guidelines precipitated blanket restrictions of opioid prescribing practices to previously opioid-naïve patients, including limitations to supply and dose,^3^^,^^4^ leading to a heightened concern over undertreatment of patient pain.^5^^,^^6^ Data-driven clinical decision support (CDS) systems have the potential to provide more individualized assessments of patients’ risk of new long-term opioid use that, when incorporated into clinical judgment, can support more nuanced prescribing decisions for patients newly started on opioids.

Prescription drug monitoring programs (PDMPs) are state-run controlled substance prescribing programs that track all outpatient opioids dispensed in their respective areas. Prescription drug monitoring programs are active throughout the United States with similar programs in Australia,^7^ Canada,^8^ and South Korea.^9^ Typically, PDMPs include web or electronic medical record interfaces for clinical review of an individual patient’s controlled substance prescribing history, which make PDMPs suitable data sources for developing and implementing CDS. However, with minimal resources^10^ and staffing,^11^ some PDMP agencies have adopted a controversial^12^^,^^13^ proprietary CDS system (NarxCare) with questionable performance,^14^ limited transparency,^15^ and concerns over model bias.^16–19^ As a result, clinical PDMP users that rely upon this tool to make prescribing decisions risk exposing themselves to legal jeopardy.^20^ State agencies that manage PDMPs would better serve their state residents by having increased control over well-tested CDS, thus allowing them the necessary flexibility to address concerns voiced by prescribers, pharmacists, and patients.

With the goal of producing a fully transparent and accessible predictive modeling approach, we previously developed and validated a PDMP-derived risk prediction model to predict new long-term opioid use in California.^21^ To mimic development scenarios where PDMP agencies would have only the data provided by pharmacies, we avoided patient predictors and outcomes (eg, overdose) that would require linkage to outside data sources. Furthermore, the model used a traditional logistic regression approach and avoided newer machine learning (ML) methodologies that are not necessarily accessible to PDMP data analysts. While the California model had high discrimination and was well-calibrated, it remained unclear how well it would perform in other states. As a critical next step in CDS development, validation studies are necessary to ensure model transportability to different settings and populations. Despite substantial efforts to develop opioid-related prediction models, few have been subject to validation studies.^19^^,^^22^

To build on our prior work, we conducted an external validation of this California-based model by applying it to Kentucky PDMP records. To further assess the performance of the California model, we compared its performance to an array of Kentucky-specific ML models. We hypothesized that when applied to Kentucky PDMP records, and with minimal updates to the model, the updated California model would perform comparably to Kentucky-specific ML models. If the updated California model could reach the performance levels of the Kentucky-specific models, it would indicate potential for use of this California model as a foundation by which the feasibility for PDMP agencies in other states to develop their own CDS systems would be significantly improved.

## Methods

### Compliance with ethics and reporting guidelines

Study reporting follows the Transparent Reporting of a multivariable prediction model for Individual Prognosis or Diagnosis (TRIPOD) guidelines for reporting multivariable prediction models.^23^ The study was approved by the University of Kentucky Institutional Review Board. The content is solely the responsibility of the authors and does not necessarily represent the official views of the Kentucky Cabinet for Health and Family Services, Office of Inspector General. The National Institutes of Health supported this study but had no role in its design, conduct, or reporting.

### Data source

De-identified, patient-level prescription data from 2019 to 2023 for all patients in Kentucky aged ≥12 were obtained from Kentucky’s PDMP. Data included details of all outpatient Schedule II-V controlled substance prescriptions dispensed to opioid-exposed patients in the state from physicians, retail pharmacies, or via internet or mail order pharmacies to a Kentucky resident. Prescription drugs were identified using National Drug Codes.^24^ Encrypted patient, prescriber, and pharmacy identifiers were included to allow tracking of these individual entities over time. Incomplete records were excluded from analysis. Prescription drug monitoring program records from 2021 to 2022 were used to create a model development patient cohort, while records from 2023 were used to create a model validation patient cohort.

### Participants

The study cohort comprised all opioid-naïve persons prescribed an index solid oral dosage (referred to as “pill”) form opioid analgesic in Kentucky between 2021 and 2023 (Figure 1). Patients were considered opioid-naïve if they were not dispensed opioid prescriptions in the previous 2 years. Records from 2019 to 2020 were used to verify patients’ opioid-naïve status. Patients who exclusively received non-pill opioids on the index day were excluded from analysis as they likely represented special cases (eg, liquid opioids to a patient who cannot swallow pills) where predictions of long-term use would be of less clinical value. Patients with prescriptions for buprenorphine formulations indicated for the treatment of opioid use disorder, as an index prescription or any time in the 30 days following the index prescription date, and/or who received a daily dose of ≥500 morphine milligram equivalents (MMEs) on their index day were excluded; such patients are unlikely to be truly opioid-naïve.^25^ Prescriptions for opioid-containing cough medications and intravenous, intramuscular, and compound opioid formulations were excluded. Criteria for participant inclusion are identical to those used in the previously published California model.^21^

**Figure 1.**
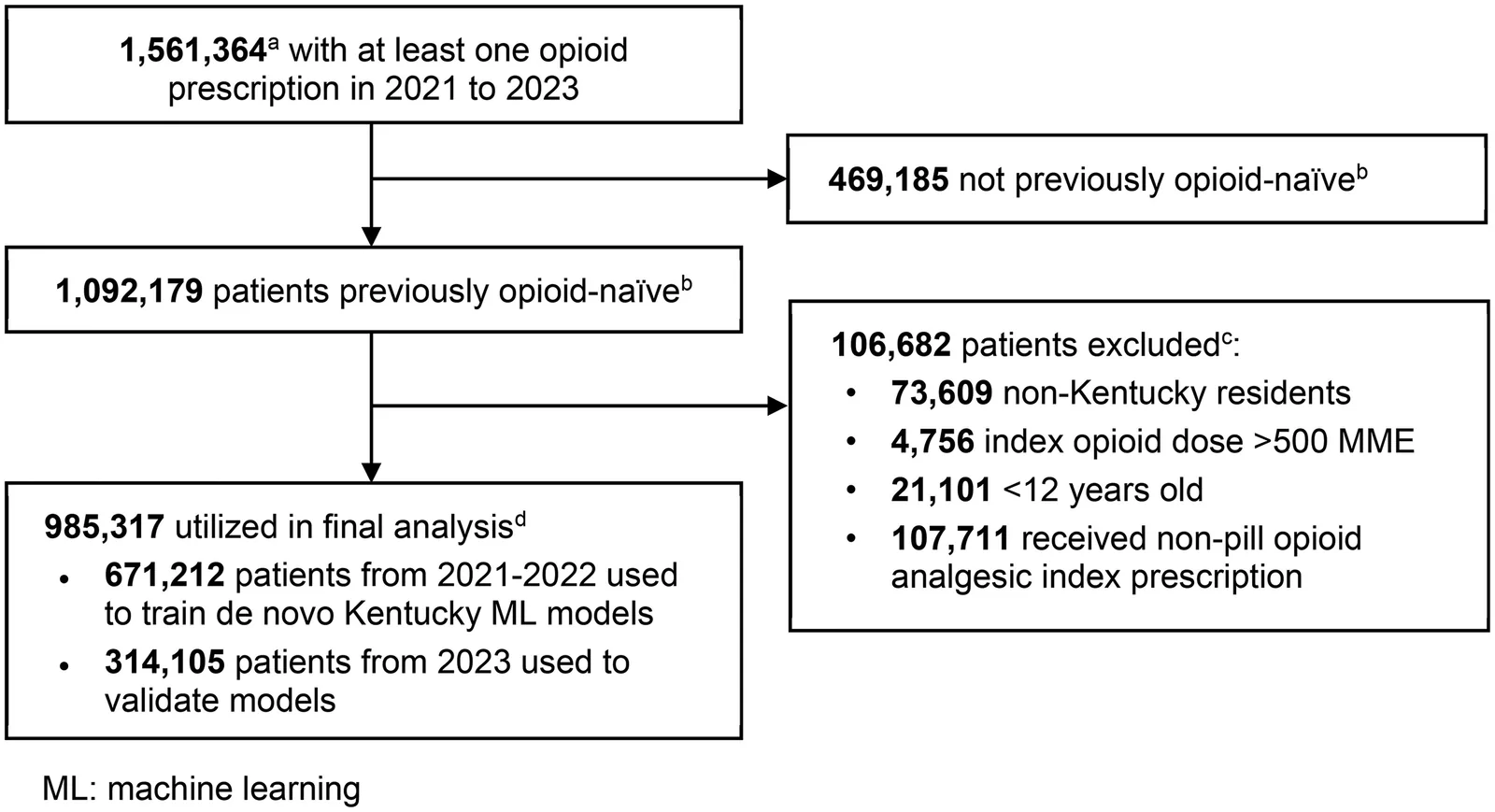
Participant flowchart through the study. ^a^With complete patient records. ^b^Defined as a patient receiving a new opioid analgesic prescription with no other opioid prescribed in the previous 730 days, and no opioid indicated for treatment of opioid use disorder prescribed in the index prescription or follow-up period. ^c^Individual patients may meet multiple exclusion criteria. ^d^Some patients (*n* = 14 882) qualify as opioid-naive in multiple years due to at least 730 days between their opioid use episodes and therefore appear in both the development and validation datasets. Abbreviation: ML, machine learning.

### Outcome

Long-term use was defined as an opioid prescription episode that lasted longer than 90 days, contained at least 3 opioid prescriptions, and had no more than 60 days between the completion of 1 prescription and the initiation of the subsequent one. This outcome is identical to that used in the previously published California model.^21^

### Predictors

Predictors were constructed using PDMP prescription records from the first 30 days post index prescription. Predictors used in the updated California model are detailed in prior work^21^ and include patient demographics (age, sex), opioid-related variables (type(s) of opioid in the index prescription; total unique pharmacies, prescribers, prescriptions per prescriber, pills per prescription from the index date through day 30; active liquid opioid formulations on day 30, active nonpill and nonliquid opioid formulations on day 30), active non-opioid controlled substance prescriptions on day 30 (benzodiazepines, other controlled substances), and opioid dose trajectory measures based on smoothed polynomial summary measures (day where dose curve maximum occurs, the total area under the dose curve, change in area under the dose curve between the first and second 10-day periods of follow-up). Details on smoothed dose trajectory curve construction and derived variables can be found in the supplement (eAppendix 1: details of opioid trajectory variable construction). Additional candidate predictors were considered in the building of Kentucky ML models. A table of these candidate predictors can be found in the supplement (eTable 1: candidate predictors considered for model development).

### Statistical analysis

Study analyses followed 2 stages. The first stage involved an external validation of the previously published California model^21^ utilizing the Kentucky 2023 validation cohort. The predictions generated were then updated using “logistic calibration”^26^ which involves adjusting the intercept and slope of the linear predictor (the logit-transformed linear combination of the model’s independent variables and their corresponding regression coefficients).^27^ Updated predictions were used to assess model performance on the Kentucky validation cohort.

The second stage involved the development of multiple novel Kentucky-based prediction models using ML algorithms commonly used in healthcare research^28^: least absolute shrinkage and selection operator (LASSO) logistic regression, elastic net logistic regression, deep neural network (DNN), random forest, and extreme-gradient boosting (XGBoost). Kentucky models were developed using the 2021 and 2022 Kentucky cohort and performance metrics were based on predictions on the 2023 Kentucky validation cohort. Model development utilized automated feature selection based on the pool of candidate predictors described above. A detailed description of the development of Kentucky ML models can be found in the supplement (eAppendix 2: development of Kentucky ML models). Performance of the updated California model and the Kentucky ML models were compared using various measures of discrimination (sensitivity, specificity, negative and positive predictive values, accuracy, area under the receiver operating characteristic curve [AUROC], normalized Matthew’s correlation coefficient [nMCC]) and calibration curves. Calibration intercepts and slopes were estimated using logistic recalibration of the observed outcome on the logit of predicted probabilities, with 95% confidence intervals (CIs). Calibration-in-the-large was estimated with the calibration slope fixed at 1. As random forest-derived predictions included exact probabilities of 0 and 1, a continuity correction to predicted probabilities was applied to calculate calibration slope and intercept estimates. Additionally, we conducted a sensitivity analysis to evaluate model performance on modified cohorts excluding the small number of individuals with separate opioid-naïve episodes in both the model development and validation Kentucky cohorts (*n* = 14 882).

All analyses were conducted using SAS 9.4 and R (version 4.4.2).

## Results

### Patient characteristics

Between 2021 and 2023, 63.1% (985 317) of Kentucky residents who filled at least 1 opioid prescription (1 561 364) met our definition for previously opioid-naïve status (Figure 1). Among these patients, 33 298 (3.4%) transitioned to new long-term opioid use—24 318 (3.6%) in the development cohort and 8980 (2.9%) in the validation cohort. Patient and prescription characteristics across the 2 cohorts were similar (Table 1).

**Table 1.** Characteristics of previously-opioid naïve patient cohorts used in model development and validation.

Variable	Development cohort 2021-2022 (*n* = 671 212) Median (IQR) or *n* (%)		Validation cohort 2023 (*n* = 314 105) Median (IQR) or *n* (%)	
	No LTU^a^ (*n* = 646 894)	LTU (*n* = 24 318)	No LTU (*n* = 305 125)	LTU (*n* = 8980)
**Patient characteristics**				
**Male sex**	286 011 (44.2%)	11 058 (45.5%)	134 999 (44.2%)	4075 (45.4%)
**Patient age**	48 (31, 64)	61 (50, 71)	48 (32, 64)	61 (51, 72)
**Opioid in index prescription**				
**Hydrocodone**	302 957 (46.8%)	8587 (35.3%)	153 334 (50.3%)	3595 (40.0%)
**Oxycodone**	160 485 (24.8%)	4178 (17.2%)	89 480 (29.3%)	1648 (18.4%)
**Tramadol**	55 242 (8.5%)	6631 (27.3%)	21 055 (6.9%)	1887 (21.0%)
**Codeine**	23 044 (3.6%)	792 (3.3%)	9051 (3.0%)	252 (2.8%)
Other^b^	105 166 (16.3%)	4130 (17%)	32 205 (10.6%)	1598 (17.8%)
**Prescription characteristics during 30-day post index period^c^**				
**Two or more prescribers**	55 207 (8.5%)	6931 (28.5%)	25 369 (8.3%)	2513 (28.0%)
**Two or more pharmacies**	30 443 (4.7%)	3512 (14.4%)	15 393 (5.0%)	1274 (14.2%)
**Mean pills per prescription**	15.0 (12.0, 20.0)	36.0 (22.0, 60.0)	15.0 (12.0, 20.0)	36.7 (22.0, 60.0)
**Mean prescriptions per prescriber**	1 (1, 1)	1 (1, 2)	1 (1, 1)	1 (1, 2)
**Change in total pill quantity on day 30 vs day 1**	−15 (−20, −12)	0 (−18, 21)	−12 (−20, −10)	0 (−16, 21)
**Active prescriptions on day 30**				
**Long-acting opioid**	26 (0.0%)	65 (0.3%)	12 (0.0%)	16 (0.2%)
**Liquid opioid**	3214 (0.5%)	197 (0.8%)	1752 (0.6%)	84 (0.9%)
**Non-liquid non-pill opioid**	35 258 (5.5%)	5088 (20.9%)	17 284 (5.7%)	1839 (20.5%)
**Benzodiazepine**	12 016 (1.9%)	1595 (6.6%)	5320 (1.7%)	572 (6.4%)
**Non-opioid Non-benzodiazepine controlled substance**	58 643 (9.1%)	7419 (30.5%)	28 485 (9.3%)	2658 (29.6%)
**Dose trajectory characteristics^c^^,^^d^**				
**Total AUdC**	13.7 (10.1, 25.3)	58.0 (29.0, 87.0)	13.0 (10.1, 22.6)	58.0 (29.8, 87.0)
**Change in AUdC from days 1-10 to days 11-20**	−16.0 (−21.0, −11.7)	−5.1 (−17.0, 0.0)	−14.0 (−20.2, −11.7)	−3.7 (−15.3, 0.0)
**Day of maximum dose**	0.0 (0.0, 0.0)	4.7 (0.0, 29.0)	0.0 (0.0, 0.0)	6.6 (0.0, 29.0)

a Long-term use defined as an episode of opioid use lasting >90 days after the initial prescription, including at least 3 opioid prescriptions, with no more than a 60-day gap between prescriptions.

b Includes patients with opioid types not specified in other categories and/or those patients who filled more than 1 type of opioid on the index day.

c Only opioid prescriptions for pill formulations (eg capsules, tablets, pills) were used to calculate these variables.

d Variables derived from cubic polynomial curves fit to raw patient daily quantity totals. Curves were fit to each individual patient for their daily dosage (quantity) from the first day of their index prescription through day 30.

Abbreviations: AUdC, area under the smoothed dose curve; LTU, long-term use.

Bivariate comparisons by transition to new long-term use revealed some noticeable differences. Patients who transitioned to new long-term use were older (median [IQR], 61 [50, 71] vs 48 [31, 64]), more likely to be prescribed tramadol (25.6% vs 8.0%), more likely to have 2 or more prescribers (28.4% vs 8.5%) and pharmacies (14.4% vs 4.8%), had a higher mean pill count in their prescriptions (median [IQR], 36.0 [22.0, 60.0] vs 15.0 [12.0, 20.0]), had a smaller change in total pill quantity between the index day and day 30 post index (median [IQR], 0 [−18, 21] vs −14 [−20, −10]), and were more likely to have an active prescription for non-liquid non-pill opioids (20.8% vs 5.5%), benzodiazepines (6.5% vs 1.8%), and other (non-opioid non-benzodiazepine) controlled substances (30.3% vs 9.1%). Additionally, dose trajectory measures indicated that total area under the smoothed dose curve (median [IQR], 58.0 [29.0, 87.0] vs 13.5 [10.1, 24.6]) and day of maximum dose (median [IQR], 4.7 [0.0, 29.0] vs 0.0 [0.0, 0.0]) were greater for opioid-naïve patients who transitioned to new long-term use.

### Model results—updated California model

Performance of the 2018 California model updated using 2023 Kentucky data is presented in Table 2.

**Table 2.** Model performance metrics, based on decision cutoffs as calculated by maximizing the Youden’s J statistic.^a^

Algorithm	Development data	Validation data	Decision cutoff^b^	Cutoff%^c^	Acc	Sens	Spec	PPV	NPV	AUROC	nMCC
**Original MLR** ^d^	CA, 2016-2017	CA, 2018	0.049	81.4%	0.846	0.844	0.848	0.213	0.991	0.913 (0.912-0.914)	0.688
**Updated MLR**	CA, 2016-2018	KY, 2023	0.030	85.0%	0.872	0.829	0.873	0.183	0.993	0.919 (0.915-0.923)	0.676
**LASSO**	KY, 2021-2022	KY, 2023	0.026	83.4%	0.858	0.861	0.858	0.171	0.994	0.925 (0.921-0.929)	0.673
**Elastic Net**	KY, 2021-2022	KY, 2023	0.026	83.3%	0.858	0.860	0.858	0.172	0.994	0.925 (0.921-0.929)	0.673
**DNN**	KY, 2021-2022	KY, 2023	0.030	85.0%	0.872	0.846	0.873	0.186	0.994	0.923 (0.920-0.927)	0.680
**Random Forest**	KY, 2021-2022	KY, 2023	0.005	82.4%	0.845	0.820	0.846	0.154	0.993	0.872 (0.866-0.877)	0.656
**XGBoost**	KY, 2021-2022	KY, 2023	0.031	84.9%	0.872	0.859	0.872	0.187	0.995	0.930 (0.926-0.933)	0.682

a Youden’s J calculation = Sensitivity + Specificity − 1.

b Represents an exploratory model decision threshold, determined by maximizing the model’s Youden’s J calculation, above which point individual predictions with a probability greater than the cutoff are predicted as having new long-term opioid use.

c Proportion of validation sample below cutoff.

d Metrics from Tseregounis et al. A risk prediction model for long-term prescription opioid use. Med Care. 2021.

Abbreviations: Acc, accuracy; AUROC, area under the receiver operating characteristic curve; CA, California; DNN, deep neural network; KY, Kentucky; LASSO, least absolute shrinkage and selection operator regression; MLR, non-penalized multiple logistic regression; nMCC, normalized Matthew’s correlation coefficient; NPV, negative predictive value; PPV, positive predictive value; Sens, sensitivity; Spec, specificity; XGBoost, extreme gradient boosting.

Model discrimination was excellent with an AUROC (95% CI) of 0.919 (0.915-0.923). At an exploratory model threshold—identified by maximized Youden’s J calculations—a high negative predictive value and low positive predictive value were observed. Model calibration is presented in Figure 2, with the calibration intercept (0.010, 95% CI: [−0.021, 0.041]) and slope (0.997, 95% CI: [0.979, 1.014]) close to the ideal values of 0 and 1, respectively. Results were comparable to Kentucky ML models.

**Figure 2.**
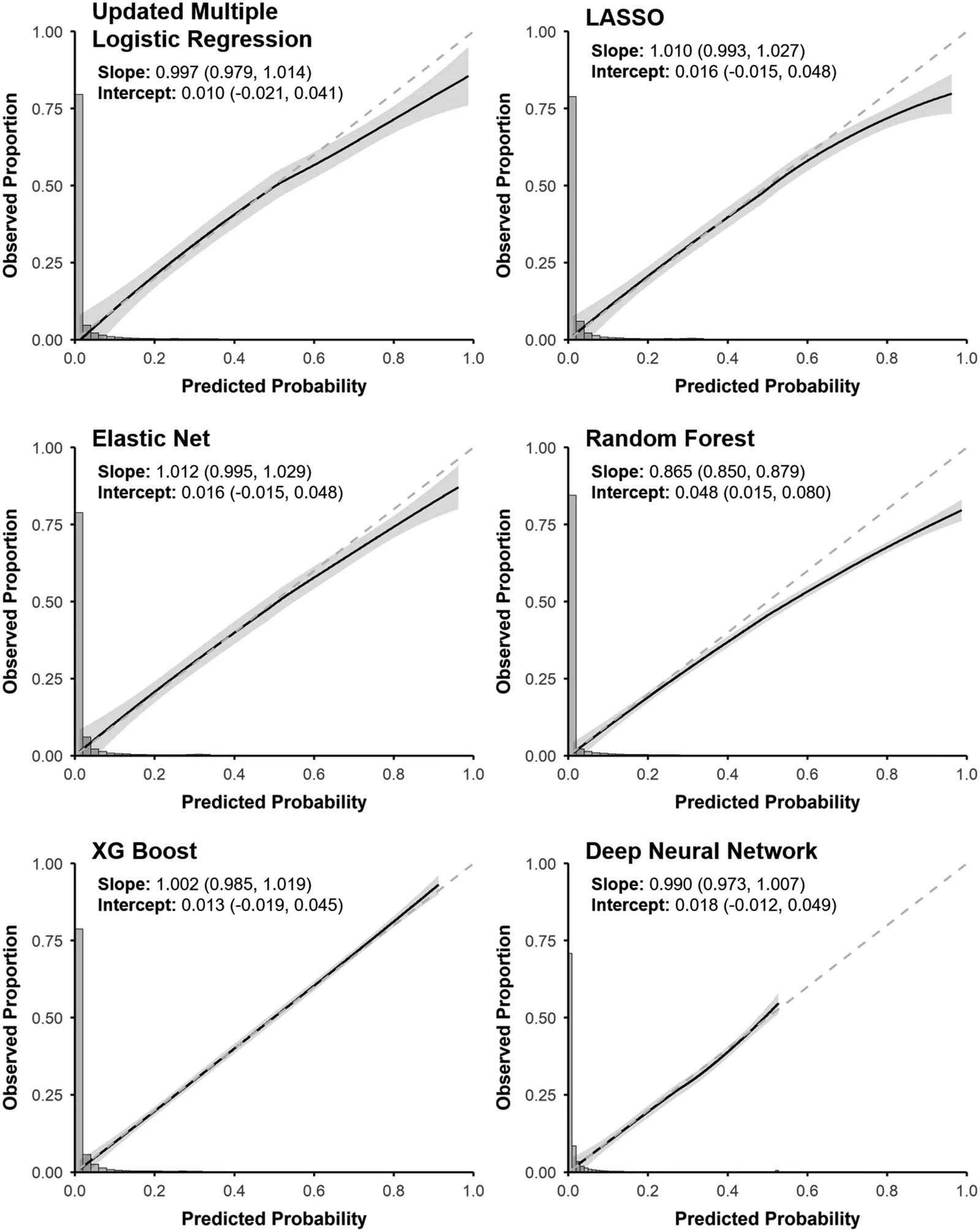
Calibration curves for models predicting new long-term opioid use among Kentucky residents, 2021-2023. The plots show mean observed outcome (new long-term use) by deciles of predicted probability (represented by the markers in the plots). Predicted probabilities are from application of the individual models, either developed on 2016-2018 California data and updated using KY 2023 data (“Updated”) or developed on 2021-2022 Kentucky data, to a validation cohort consisting of 2023 Kentucky data. The dashed gray line reflects perfect agreement between observed and predicted probabilities. The solid line represents a Loess smoothed calibration curve fit across probability deciles, restricted to a range of probabilities supported by the predictions, with the shaded gray bands representing 95% CIs. The bars along the *x*-axis represent the distribution of predicted probabilities in the test set, illustrating the concentration of predictions across the probability range.

### Model results—Kentucky models

Model discrimination was consistently good to excellent for all developed models with AUROC values ranging from 0.872 (95% CI: 0.866, 0.877) for random forest to 0.930 (95% CI: 0.926, 0.933) for XGBoost. When comparing all other measures of discrimination at the probability thresholds determined by maximized Youden’s J, XGBoost was consistently the highest performing model while random forest was the worst performing. Calibration performance was comparable to the updated California model for all models except random forest (Figure 2), with an apparent overestimation of risk at higher levels (calibration slope: 0.865, 95% CI: [0.850, 0.879]; calibration intercept: 0.048, 95% CI: [0.015, 0.080]).

Sensitivity analyses showed no meaningful change in model performance when trained and validated on the modified Kentucky cohorts that excluded individuals with separate opioid-naïve episodes in both the development and validation cohorts (eTable 2: sensitivity analysis comparing model performance with models developed and validated excluding all individuals who contribute observations to both development and validation cohorts).

## Discussion

In this study, we externally validated a California PDMP-based prediction model for new long-term opioid use using Kentucky PDMP records. Following a simple logistic calibration update, the model demonstrated excellent discrimination and was well-calibrated up to the highest risk levels, results that indicate good model transportability. Upon comparison to new Kentucky models, the updated model’s performance holds up well with observed deficits in performance of the updated model offset by gains in feasibility for CDS development.

As observed in our previous work,^21^ PDMP prediction models demonstrated an excellent capacity to rule out new long-term opioid use for patients at the lower end of the risk spectrum. Models had negative predictive values consistently higher than 99% and were well-calibrated up to the highest risk levels. Alternatively, the models all consistently struggled with low positive predictive values (17%-19%), indicating that any predictions for new long-term use are likely to be false positives. These findings reveal potential clinical utility for ruling out new long-term use in patients where long-term use of opioids is neither expected nor clinically appropriate. Subsequent CDS systems could improve prescriber confidence in prescribing patients experiencing acute pain by refining clinical risk assessment; improving pain management outcomes among low-risk patients while also minimizing concerns over risk for unneeded patient transition to long-term opioid use.

Our CDS tool incorporates predictors from the first 30 days of incident opioid use and is therefore most clinically useful for identifying previously opioid-naïve patients who were recently started on opioids for acute pain (≤30 days) and may be at risk of transitioning to chronic/long-term opioid use (≥90 days). For example, when evaluating a patient who was started on opioids 30 days prior, a clinician who sees the patient is at moderate or higher risk for transitioning to long-term opioid use may decide to reassess the patient, ensuring that potentially reversible causes of chronic pain have been addressed and that decisions to continue opioids are made intentionally. Therefore, this tool, by alerting clinicians to patients at higher risk of transitioning to long-term opioid use, may thereby reduce the number of patients who transition from acute to chronic/long-term use due to clinical inertia rather than an intentional clinical decision—a clinical goal aligned with the 2022 CDC opioid guideline recommendations to ensure that “opioid prescribing for acute pain does not unintentionally become long-term opioid therapy simply because medications are continued without reassessment.”^2^

This study’s findings have implications for future PDMP CDS development. Ideally, managing agencies would be able to develop CDS systems in-house while working together with clinical and community stakeholders to ensure effective implementation with minimal harm to patients. To date, opioid-related CDS development has either occurred outside the PDMP domain, such as Epic System’s Opioid Risk Score^29^ and the Veterans Health Administration’s Stratification Tool for Opioid Risk Mitigation,^30^ or has been limited to simple individual variable CDS alerts. For more robust CDS, PDMPs mostly rely on a third-party developer, Bamboo Health, that offers a product (NarxCare) with concerns regarding model external validity, prediction quality, and clinical utility. As such, deployment of this third-party tool has been the subject of significant skepticism and criticism.

Utilization of the 2018 California model as a foundation can greatly improve the feasibility for agencies to develop their own CDS systems for ruling out new long-term use among previously opioid-naïve patients. The model development process requires careful data cleaning and preprocessing, followed by complex data manipulation, variable construction, and model development, steps that require developers to have access to technical expertise in CDS system development alongside extensive clinical knowledge and opioid prescribing experience. For example, developing the previous 2018 California model required over a year of effort from members of a multidisciplinary study team consisting of multiple statisticians, epidemiologists, programmers, and clinicians. In this study, Kentucky ML model development was lengthy even when using all of the pre-processing and variable construction work completed for the 2018 California model to fast-track the development process; requiring a month of work by a trained data scientist consulting with experienced epidemiologists. For a PDMP agency to conduct all that work from scratch would require an amount of time, experience, and effort that is not typically available without substantial outside personnel and funding.

Alternatively, this foundational model could allow agencies to forego those steps and begin at the very end of the development process. In the update process of the California model, we were able to adapt code to create the final dataset, run the model update step, and evaluate performance measures in a single week. With similar access to prepared prescription record data, which PDMP agencies already possess, and the necessary code, which is publicly available via code links in the supplement, agencies can reproduce a similar prediction model using their own data. Thus, this provides a substantial boost to feasibility for in-house PDMP CDS development and saves resources for necessary downstream implementation steps.^31^

Prescription drug monitoring program-based models, and by extension PDMP-based CDS systems, have limitations. First, prescription records are limited by the data outpatient pharmacies are required to provide and may vary by state. Second, most PDMPs only capture pharmacy records from their states; states with large border regions and/or transient populations may be most impacted as the data will not capture controlled substance prescribing from areas outside the state. Third, the false positive rate for PDMP-based models is high. This limitation was also found in other PDMP-derived models developed to predict overdose,^32–34^ partly due to a combination of a low outcome prevalence (3.4% in this study) and unavailable patient information (PDMP records lack diagnostic and clinical data). Therefore, these models should not be relied upon to make patient-centered decisions but may instead be useful as supplementary tools to aid providers in prescribing decisions where they can weigh model predictions alongside key clinical patient factors. Finally, logistic calibration of the California model was estimated using the entire validation cohort, also used to calculate performance metrics, which may add optimism to model performance; however, this concern is mitigated by the high events per variable in the validation cohort (>500), minimizing the magnitude of any potential optimism.

In conclusion, our study demonstrated that a model developed to predict new long-term opioid use in California could be updated to predict new long-term use in Kentucky with a high degree of discrimination and good calibration. This transportable model has performance characteristics comparable to ML models and can act as a foundational instrument to catalyze CDS system development by PDMP agencies to support identification of patients at relatively low predicted risk. Future research evaluating the clinical utility of these models and a prospective impact assessment of their implementation are necessary.

## Supplementary Material

ooag192_Supplementary_Data

## Data Availability

Due to privacy and data use agreement restrictions, individual-level PDMP data used for this study cannot be made available. Study code is available at the following 2 links: https://github.com/etseregounis/SAS-PDMP-CDS-Code; https://github.com/eugene-shin-ds/Predicting-Long-Term-Opioid-Use-from-PDMP-Data/tree/main
