# Supplementary material for: Evaluating the validity and performance of a modifiable prediction model to predict new long-term opioid use in prescription drug monitoring programs
Source: JAMIA Open. 2026 Sep 30;9(5):ooag192. doi: 10.1093/jamiaopen/ooag192 (PMC13625009; doi:10.1093/jamiaopen/ooag192)
Supplement: Supplementary file 1 [file ooag192_supplementary_data.docx]

**Supplementary Materials**

Evaluating the validity and performance of a modifiable prediction model to predict new long-term opioid use in prescription drug monitoring programs

| **Table of Contents** | |
| --- | --- |
| **Page** | **Content** |
| **1** | **Table of Contents and Code Links** |
| **2** | **eAppendix 1:** Details of opioid trajectory variable construction |
| **6** | **eTable 1:** Candidate predictors considered for model development |
| **11** | **eAppendix 2:** Development of Kentucky machine learning models |
| **13** | **eTable 2:** Sensitivity analysis comparing model performance with models developed and validated excluding all individuals who contribute observations to both training and validation cohorts |
|  |  |
| **Code Links** | |
| SAS code to update PDMP data, construct predictors, and update and validate model:  [**https://github.com/etseregounis/SAS-PDMP-CDS-Code**](https://github.com/etseregounis/SAS-PDMP-CDS-Code) | |
| R code to develop machine learning models:  [**https://github.com/eugene-shin-ds/Predicting-Long-Term-Opioid-Use-from-PDMP-Data/tree/main**](https://github.com/eugene-shin-ds/Predicting-Long-Term-Opioid-Use-from-PDMP-Data/tree/main) | |

**eAppendix 1:** Details of opioid trajectory variable construction

In the development of models that predict the risk of opioid naïve patients transitioning to long-term (>90-day) use, we explored the predictive value of 30-day post-index dose trajectories. The nature of the data allowed us to identify all opioid prescriptions for each individual patient from the start of their index prescription to the 30^th^ day of follow-up. Each prescription had two versions of dose calculated: prescription quantity, using the following calculation:

*Eq.1*	$Daily Quantity=\frac{(Quantity)}{(Days Supply)}$

and Milligram Morphine Equivalents (MME), calculated using the following equation:

*Eq.2*	$Daily MME=\frac{\left( Strength per Unit \right)*(Quantity)*(Conversion Factor)}{(Days Supply)}$

Overlapping prescriptions’ daily dosages were totaled to determine each patient’s daily dosage in the follow-up period. We were interested in evaluating daily totals of dose as well as how these totals deviated from a sample average, from here onwards referred to as residual values. Daily residuals were determined using PROC GAMPL procedure in SAS 9.4. It is not uncommon for subsequent prescriptions in a patient’s episode to be filled ‘early’ (e.g. before the run-out data of the prior prescription) and create spikes in the dosage trajectories. To account for this noise, we smoothed the daily totals and residuals, using the PROC REG procedure in SAS 9.4, by fitting these values to the following third-order polynomial:

*Eq.3*	$Y_{i}=\beta_{3i}\left( D^{3} \right)+\beta_{2i}\left( D^{2} \right)+\beta_{1i}\left( D \right)+\beta_{0i}$

Where $i$ denotes the $i^{th}$ subject, $D$ refers to day post index prescription fill, and $Y$ refers to either total dose or the residual. *Fig.1* is an example of a previously opioid-naïve

*Fig.1*

patient’s 30-day dose trajectory showing the raw daily dosage (in pill quantity) relative to a smoothed cubic polynomial fit to the raw totals. Restricted cubic splines were considered as an alternative smoothing technique but comparisons of the two methods showed similar results. *Fig.2* shows separate plotted 30-day trajectories for five different patients; the points represent raw daily quantity totals, the solid line a cubic polynomial fit to the raw totals, and the dashed line a restricted cubic spline fit (with four knots) to the raw totals.

*Fig.2*

These individual smoothed dosage trajectories now formed the basis for the generation of summary measures that could be used to characterize patient patterns in dose. First, we calculated the area under the curve for each patient’s trajectory. These were determined by taking the integral of *Eq.3*, inputting the patient-specific coefficients, and solving for the limits, in this case days following the index fill, as desired.

*Eq.4*	${AUC}_{i, a,b}=\int_{a}^{b} (Eq.3)dD=\left[ \frac{\beta_{3i}\left( D^{4} \right)}{4}+\frac{\beta_{2i}\left( D^{3} \right)}{3}+\frac{\beta_{1i}\left( D^{2} \right)}{2}+\beta_{0i}(D) \right]_{a}^{b}$

Where $i$ denotes the $i^{th}$ subject,$a$ and $b$ refer to the first and last day of the period in question respectively, and $AUC$ refers to area under the smoothed curve.

Additionally, we determined local critical points (maximum, minimum) in the follow-up period by taking the derivative of *Eq.3*, setting it equal to 0, and then solving for the day on which critical points occurred ($D_{c}$) using the quadratic formula.

*Eq.5*	$\frac{dY_{i}}{dD}={3\beta}_{3i}\left( D^{2} \right)+{2\beta}_{2i}\left( D \right)+\beta_{1i}$

*Eq.6*	$D_{c}=\frac{-\beta_{2i} \pm\sqrt{{(\beta_{2i})}^{2}-3(\beta_{3i})(\beta_{1i})}}{3\beta_{3i}}$

Values were determined by inputting the solved-for day back into *Eq.3*. Days with the lowest and highest values, either occurring within the 30-day follow-up period, on the first day, or on the last day, were considered the minimum and maximum dosage (or residual) days respectively.

Finally, we determined days on which the greatest 10-day increase in dose or residual occurred. This increase ($C_{i}$) is shown in the following expression:

*Eq.7*	$C_{i}=\left[ \beta_{3i}\left( D^{3} \right)+\beta_{2i}\left( D^{2} \right)+\beta_{1i}\left( D \right)+\beta_{0i} \right]-$

$$[\beta_{3i}\left( D-10 \right)^{3}+\beta_{2i}\left( D-10 \right)^{2}+\beta_{1i}(D-10)+\beta_{0i}]$$

Which can be simplified to:

*Eq.8*	$C_{i}=\left( 30\beta_{3i} \right)D^{2}+\left( -300\beta_{3i}+20\beta_{2i} \right)D+(1000\beta_{3i}-100\beta_{2i}+10\beta_{1i})$

To determine the day of maximum increase we took the derivative of *Eq.8*, set it equal to 0, and then solved for the day on which the critical point occurred.

*Eq.9*	$\frac{dC_{i}}{dD}={60\beta}_{3i}\left( D \right)-300\beta_{3i}+20\beta_{2i}$

*Eq.10*	$D_{c}=\frac{-\beta_{2i}}{3\beta_{3i}}+5$

Values were determined by inputting the solved-for day back into *Eq.8*. We then compared this critical value to the increases occurring between days 0-10 and days 20-30. The largest value represented the day on which the greatest 10-day increase occurred. A visual example of how these variables are interpreted can be found in *Fig.3* below:

*Fig.3*

The shaded area represents the area under the smoothed curve (AUC). The dashed line in blue denotes the cutoff for the first ten days and the enclosed AUC for that time period. The black arrows point to the local maximum and minimum.

**eTable 1:** Candidate predictors considered for model development

| **Predictor Category** | **Predictor** | **Type** | **Values/Notes** |
| --- | --- | --- | --- |
| Patient demographics | Patient sex | Binary | Female, Male |
|  | Patient age | Continuous |  |
| Index opioid | Year of episode | Categorical | 2021, 2022, 2023 |
|  | Total opioid pills | Continuous | Total pills prescribed on index day |
|  | Total MME |  | Total MME prescribed on index day |
|  | Opioid type | Categorical | Hydrocodone, Oxycodone, Tramadol, Codeine, Other |
| 30-Day period post-index | Total pills | Continuous | Total opioid pills prescribed in first 30 days of episode |
|  | Opioid pill quantity per prescription | Continuous | Total opioid pills prescribed divided by total opioid pill prescriptions |
|  | Opioid pill MME per prescription | Continuous | Total pill opioid MME prescribed divided by total opioid pill prescriptions |
|  | Opioid prescriptions per prescriber | Continuous | Total pill opioid prescriptions divided by total unique prescribers of pill opioids |
|  | Total opioid prescribers | Binary | 1, 2 or more |
|  | Total opioid prescriptions | Binary | 1, 2 or more |
|  | Total opioid MME | Continuous |  |
|  | Total opioid pill quantity | Continuous |  |
|  | Total long-acting opioid prescriptions | Continuous |  |
|  | Change in pill quantity | Continuous | Number of opioid pills on Day 30 minus the number of pills on index day |
|  | Change in MME |  | Total MME on Day 30 minus total MME on index day |
| Day 30 post-index | Active benzodiazepine | Binary | Indicator if patient had at least one active benzodiazepine prescription |
|  | Active long-acting opioid | Binary | Indicator if patient had at least one active long-acting opioid prescription |
|  | Active liquid opioid | Binary | Indicator if patient had at least one active liquid opioid prescription |
|  | Active non-pill non-liquid opioid | Binary | Indicator if patient had at least one active non-pill non-liquid opioid prescription |
|  | Active other controlled substance | Binary | Indicator if patient had at least one active non-opioid non-benzodiazepine controlled substance prescription |
|  | Total pills | Continuous | Total pills prescribed on Day 30 |
|  | Total MME | Continuous | Total MME prescribed on Day 30 |
| Smoothed dose curve variables | Total quantity AUC | Continuous | Area under the pill quantity smoothed curve in first 30 days of episode |
|  | Index to Day 10 quantity AUC | Continuous | Area under the pill quantity smoothed curve between index and Day 10 |
|  | Index to Day 15 quantity AUC | Continuous | Area under the pill quantity smoothed curve between index and Day 15 |
|  | Day 10 to Day 20 quantity AUC | Continuous | Area under the pill quantity smoothed curve between Day 10 and Day 20 |
|  | Day 15 to Day 30 quantity AUC | Continuous | Area under the pill quantity smoothed curve between Day 15 and Day 30 |
|  | Day 20 to Day 30 quantity AUC | Continuous | Area under the pill quantity smoothed curve between Day 20 and Day 30 |
|  | Quantity AUC difference between Days 15-30 and Days Index-15 | Continuous | Difference in areas under the pill quantity smoothed curve between (area under the curve between Day 15 and Day 30) and (area under the curve between index and Day 15) |
|  | Quantity AUC difference between Days 10-20 and Days Index-10 | Continuous | Difference in areas under the pill quantity smoothed curve between (area under the curve between Day 10 and Day 20) and (area under the curve between index and Day 10) |
|  | Quantity AUC difference between Days 20-30 and Days 10-20 | Continuous | Difference in areas under the pill quantity smoothed curve between (area under the curve between Day 20 and Day 30) and (area under the curve between Day 10 and Day 20) |
|  | Quantity maximum day | Continuous | Day of maximum quantity based on smoothed quantity curve |
|  | Quantity minimum day | Continuous | Day of minimum quantity based on smoothed quantity curve |
|  | Maximum 10-day quantity change | Continuous | Last day of 10-day period with maximum change in quantity using smoothed quantity curve |
|  | Maximum 5-day quantity change | Continuous | Last day of 5-day period with maximum change in quantity using smoothed quantity curve |
|  | Total MME AUC | Continuous | Area under the pill MME smoothed curve in first 30 days of episode |
|  | Index to Day 10 MME AUC | Continuous | Area under the pill MME smoothed curve between index and Day 10 |
|  | Index to Day 15 MME AUC | Continuous | Area under the pill MME smoothed curve between index and Day 15 |
|  | Day 10 to Day 20 MME AUC | Continuous | Area under the pill MME smoothed curve between Day 10 and Day 20 |
|  | Day 15 to Day 30 MME AUC | Continuous | Area under the pill MME smoothed curve between Day 15 and Day 30 |
|  | Day 20 to Day 30 MME AUC | Continuous | Area under the pill MME smoothed curve between Day 20 and Day 30 |
|  | MME AUC difference between Days 15-30 and Days Index-15 | Continuous | Difference in areas under the pill MME smoothed curve between (area under the curve between Day 15 and Day 30) and (area under the curve between index and Day 15) |
|  | MME AUC difference between Days 10-20 and Days Index-10 | Continuous | Difference in areas under the pill MME smoothed curve between (area under the curve between Day 10 and Day 20) and (area under the curve between index and Day 10) |
|  | MME AUC difference between Days 20-30 and Days 10-20 | Continuous | Difference in areas under the pill MME smoothed curve between (area under the curve between Day 20 and Day 30) and (area under the curve between Day 10 and Day 20) |
|  | MME maximum day | Continuous | Day of maximum MME based on smoothed MME curve |
|  | MME minimum day | Continuous | Day of minimum MME based on smoothed MME curve |
|  | Maximum 10-day MME change | Continuous | Last day of 10-day period with maximum change in MME using smoothed MME curve |
|  | Maximum 5-day MME change | Continuous | Last day of 5-day period with maximum change in MME using smoothed MME curve |
| Smoothed residual dose curve variables | Quantity residual curve intercept | Continuous | Patient quantity residuals smoothed polynomial coefficient, intercept |
|  | Quantity residual curve linear term | Continuous | Patient quantity residuals smoothed polynomial coefficient, linear term |
|  | Quantity residual curve 2^nd^ order term | Continuous | Patient quantity residuals smoothed polynomial coefficient, quadratic term |
|  | Quantity residual curve 3^rd^ order term | Continuous | Patient quantity residuals smoothed polynomial coefficient, cubic term |
|  | Total quantity residual AUC | Continuous | Area under the pill quantity residual smoothed curve in first 30 days of episode |
|  | Index to Day 10 quantity residual AUC | Continuous | Area under the pill quantity residual smoothed curve between index and Day 10 |
|  | Index to Day 15 quantity residual AUC | Continuous | Area under the pill quantity residual smoothed curve between index and Day 15 |
|  | Day 10 to Day 20 quantity residual AUC | Continuous | Area under the pill quantity residual smoothed curve between Day 10 and Day 20 |
|  | Day 15 to Day 30 quantity residual AUC | Continuous | Area under the pill quantity residual smoothed curve between Day 15 and Day 30 |
|  | Day 20 to Day 30 quantity residual AUC | Continuous | Area under the pill quantity residual smoothed curve between Day 20 and Day 30 |
|  | Quantity residual AUC difference between Days 15-30 and Days Index-15 | Continuous | Difference in areas under the pill quantity residual smoothed curve between (area under the curve between Day 15 and Day 30) and (area under the curve between index and Day 15) |
|  | Quantity residual AUC difference between Days 10-20 and Days Index-10 | Continuous | Difference in areas under the pill quantity residual smoothed curve between (area under the curve between Day 10 and Day 20) and (area under the curve between index and Day 10) |
|  | Quantity residual AUC difference between Days 20-30 and Days 10-20 | Continuous | Difference in areas under the pill quantity residual smoothed curve between (area under the curve between Day 20 and Day 30) and (area under the curve between Day 10 and Day 20) |
|  | Quantity residual maximum day | Continuous | Day of maximum quantity residual based on smoothed quantity residual curve |
|  | Quantity residual minimum day | Continuous | Day of minimum quantity residual based on smoothed quantity residual curve |
|  | Maximum 10-day quantity residual change | Continuous | Last day of 10-day period with maximum change in quantity residual using smoothed quantity residual curve |
|  | Maximum 5-day quantity residual change | Continuous | Last day of 5-day period with maximum change in quantity residual using smoothed quantity residual curve |
|  | MME residual curve intercept | Continuous | Patient MME residuals smoothed polynomial coefficient, intercept |
|  | MME residual curve linear term | Continuous | Patient MME residuals smoothed polynomial coefficient, linear term |
|  | MME residual curve 2^nd^ order term | Continuous | Patient MME residuals smoothed polynomial coefficient, quadratic term |
|  | MME residual curve 3^rd^ order term | Continuous | Patient MME residuals smoothed polynomial coefficient, cubic term |
|  | Total MME residual AUC | Continuous | Area under the pill MME residual smoothed curve in first 30 days of episode |
|  | Index to Day 10 MME residual AUC | Continuous | Area under the pill MME residual smoothed curve between index and Day 10 |
|  | Index to Day 15 MME residual AUC | Continuous | Area under the pill MME residual smoothed curve between index and Day 15 |
|  | Day 10 to Day 20 MME residual AUC | Continuous | Area under the pill MME residual smoothed curve between Day 10 and Day 20 |
|  | Day 15 to Day 30 MME residual AUC | Continuous | Area under the pill MME residual smoothed curve between Day 15 and Day 30 |
|  | Day 20 to Day 30 MME residual AUC | Continuous | Area under the pill MME residual smoothed curve between Day 20 and Day 30 |
|  | MME residual AUC difference between Days 15-30 and Days Index-15 | Continuous | Difference in areas under the pill MME residual smoothed curve between (area under the curve between Day 15 and Day 30) and (area under the curve between index and Day 15) |
|  | MME residual AUC difference between Days 10-20 and Days Index-10 | Continuous | Difference in areas under the pill MME residual smoothed curve between (area under the curve between Day 10 and Day 20) and (area under the curve between index and Day 10) |
|  | MME residual AUC difference between Days 20-30 and Days 10-20 | Continuous | Difference in areas under the pill MME residual smoothed curve between (area under the curve between Day 20 and Day 30) and (area under the curve between Day 10 and Day 20) |
|  | MME residual maximum day | Continuous | Day of maximum MME residual based on smoothed MME residual curve |
|  | MME residual minimum day | Continuous | Day of minimum MME residual based on smoothed MME residual curve |
|  | Maximum 10-day MME residual change | Continuous | Last day of 10-day period with maximum change in MME residual using smoothed MME residual curve |
|  | Maximum 5-day MME residual change | Continuous | Last day of 5-day period with maximum change in MME residual using smoothed MME residual curve |

**eAppendix 2:** Development of Kentucky machine learning models

**Overview:**

All analyses were conducted in R (v4.4.2). Model development and evaluation used caret (v7.0-1) for workflow and resampling, randomForest (v4.7-1.2) for Random Forest, xgboost (v3.1.2.1) for gradient-boosted trees, glmnet (v4.1-10) for LASSO and Elastic Net, and nnet (v7.3-19) for neural network models. ROC analysis and AUC estimation were performed using pROC (v1.19.0.1). Data manipulation and reporting used dplyr (v1.1.4), tibble (v3.3.0), purrr (v1.1.0), and ggplot2 (v4.0.0), with matrix handling via Matrix (v1.7-1). Package dependencies and exact versions were managed with renv (v1.1.7) and are recorded in the project lockfile (renv.lock) to support reproducibility.

**GitHub:**

<https://github.com/eugene-shin-ds/Predicting-Long-Term-Opioid-Use-from-PDMP-Data/tree/main>

This GitHub repository contains a fully reproducible R-based machine learning pipeline developed to predict new long-term opioid use using features derived from Prescription Drug Monitoring Program (PDMP) data. The repository includes modular scripts for data preprocessing and feature engineering, training multiple models (Random Forest, XGBoost, LASSO, logistic regression variants, neural networks, and Elastic Net), and evaluating performance using discrimination, calibration, and multiple threshold optimization strategies (base, Youden, and F1). Diagnostic visualizations such as calibration plots are generated for internal model assessment, while a formal data schema and dependency lockfile (renv.lock) are provided to support transparency and reproducibility. Due to privacy, ethical, and institutional constraints associated with PDMP data, no individual-level data or quantitative results are included in the public repository.

**Methodology:**

We developed and compared multiple machine learning models to predict whether a patient would transition to long-term opioid use. The target variable was binary (long_term = 0 or 1), and we split the data into training and test sets for all experiments. Our goal was to evaluate and compare model performance using consistent procedures, including threshold optimization and multiple evaluation metrics.

We tested six models: Random Forest, XGBoost, LASSO, multivariate logistic regression (MLR), Elastic Net, and a Deep Neural Network (DNN). Each model was trained on the same training data and evaluated on the same held-out test set to ensure a fair comparison. The Youden's Index, which maximizes the sum of sensitivity and specificity, was chosen as the preferred threshold to comparatively assess model performance.

*Model Implementation and Parameters*

1. Random Forest (RF)

We used the randomForest package and trained the model with 100 trees (ntree = 100). This ensemble method creates multiple decision trees on bootstrapped samples with randomized feature selection, which helps capture complex interactions and reduce overfitting. The number of trees was chosen to balance stability and computation time.

2. XGBoost

The XGBoost model was implemented using the xgboost package with a logistic objective function. We performed 5-fold cross-validation with early stopping to find the best number of boosting rounds. Final parameters included a learning rate of 0.03, max_depth = 6, min_child_weight = 5, subsample = 0.8, colsample_bytree = 0.8, and regularization terms gamma = 0.1, lambda = 1, and alpha = 0. These were chosen to control overfitting while maintaining flexibility.

3. LASSO Logistic Regression

LASSO was fitted using the glmnet package with alpha = 1 for L1 regularization. We used 5-fold cross-validation to select the optimal lambda (lambda.min). This model helps with variable selection and reduces overfitting, especially in high-dimensional settings.

4. Elastic Net

Elastic Net was also fitted with the glmnet package, using alpha = 0.5 to combine both LASSO and Ridge penalties. As with LASSO, we used cross-validation to select the best lambda. This model is particularly useful when predictors are correlated, balancing feature selection and coefficient shrinkage.

5. Deep Neural Network (DNN)

Before training the neural network, we removed near-zero variance predictors and applied PCA for dimensionality reduction (retaining 30 components). The DNN was trained using the nnet package with one hidden layer of 5 units (size = 5) and L2 regularization (decay = 1e-4). We used a relatively small network to prevent overfitting and to keep the model simple given the transformed input.

| **eTable 2.** Sensitivity analysis comparing model performance with models developed and validated excluding all individuals who contribute observations to both training and validation cohorts | | | | | | | | | | |
| --- | --- | --- | --- | --- | --- | --- | --- | --- | --- | --- |
| **Algorithm** | **Training**  **Data** | **Validation**  **Data** | **Decision Cutoff**^a^ | **Acc** | **Sens** | **Spec** | **PPV** | **NPV** | **AUROC** | **nMCC** |
| Updated MLR | CA, 2016-18 | KY, 2023 | 0.030 | 0.872 | 0.829 | 0.873 | 0.183 | 0.993 | 0.919 (0.915, 0.923) | 0.676 |
|  | CA, 2016-18 | Ex. KY, 2023^b^ | 0.033 | 0.878 | 0.831 | 0.879 | 0.194 | 0.993 | 0.922 [0.918, 0.926] | 0.682 |
| LASSO | KY, 2021-22 | KY, 2023 | 0.026 | 0.858 | 0.861 | 0.858 | 0.171 | 0.994 | 0.925 (0.921, 0.929) | 0.673 |
|  | Ex.KY, 2021-22^b^ | Ex. KY, 2023^b^ | 0.033 | 0.881 | 0.846 | 0.882 | 0.201 | 0.994 | 0.927 [0.923, 0.931] | 0.689 |
| Elastic Net | KY, 2021-22 | KY, 2023 | 0.026 | 0.858 | 0.860 | 0.858 | 0.172 | 0.994 | 0.925 (0.921, 0.929) | 0.673 |
|  | Ex.KY, 2021-22^b^ | Ex. KY, 2023^b^ | 0.037 | 0.889 | 0.839 | 0.890 | 0.211 | 0.994 | 0.927 [0.923, 0.931] | 0.693 |
| DNN | KY, 2021-22 | KY, 2023 | 0.030 | 0.872 | 0.846 | 0.873 | 0.186 | 0.994 | 0.923 (0.920, 0.927) | 0.680 |
|  | Ex.KY, 2021-22^b^ | Ex. KY, 2023^b^ | 0.024 | 0.855 | 0.863 | 0.855 | 0.172 | 0.994 | 0.923 [0.919, 0.927] | 0.673 |
| Random Forest | KY, 2021-22 | KY, 2023 | 0.005 | 0.845 | 0.820 | 0.846 | 0.154 | 0.993 | 0.872 (0.866, 0.877) | 0.656 |
|  | Ex.KY, 2021-22^b^ | Ex. KY, 2023^b^ | 0.005 | 0.840 | 0.832 | 0.840 | 0.154 | 0.993 | 0.877 [0.872, 0.883] | 0.657 |
| XGBoost | KY, 2021-22 | KY, 2023 | 0.031 | 0.872 | 0.859 | 0.872 | 0.187 | 0.995 | 0.930 (0.926, 0.933) | 0.682 |
|  | Ex.KY, 2021-22^b^ | Ex. KY, 2023^b^ | 0.033 | 0.875 | 0.862 | 0.875 | 0.195 | 0.994 | 0.933 [0.929, 0.936] | 0.687 |
| Acc: accuracy, Sens: sensitivity, Spec: specificity, PPV: positive predictive value, NPV: negative predictive value, AUROC: area under the receiver operating characteristic curve, nMCC: normalized Matthews correlation coefficient, MLR: non-penalized multiple logistic regression, LASSO: least absolute shrinkage and selection operator regression, DNN: deep neural network, XGBoost: extreme gradient boosting, CA: California, KY: Kentucky  ^a^ Represents an optimal predictive probability, as determined by maximizing the model’s Youden’s J calculation (Sensitivity + Specificity – 1), above which point individual predictions with a probability greater than the cutoff are predicted as having new long-term opioid use  ^b^ Modified version of the cohort that excludes the 14,882 patients who appeared with separate naïve episodes in both the training and validation cohorts | | | | | | | | | | |
